# Fragmentation of outage clusters during the recovery of power distribution grids

**DOI:** 10.1038/s41467-022-35104-9

**Published:** 2022-11-30

**Authors:** Hao Wu, Xiangyi Meng, Michael M. Danziger, Sean P. Cornelius, Hui Tian, Albert-László Barabási

**Affiliations:** 1 State Key Laboratory of Networking and Switching Technology, Beijing University of Posts and Telecommunications, Beijing, 100876 China; 2 Center for Complex Networks Research, Department of Physics, Northeastern University, Boston, 02115 USA; 3 Department of Physics, Ryerson University, 350 Victoria Street, M5B 2K3 Toronto, Canada

**Keywords:** Physics, Statistical physics, thermodynamics and nonlinear dynamics

## Abstract

The understanding of recovery processes in power distribution grids is limited by the lack of realistic outage data, especially large-scale blackout datasets. By analyzing data from three electrical companies across the United States, we find that the recovery duration of an outage is connected with the downtime of its nearby outages and blackout intensity (defined as the peak number of outages during a blackout), but is independent of the number of customers affected. We present a cluster-based recovery framework to analytically characterize the dependence between outages, and interpret the dominant role blackout intensity plays in recovery. The recovery of blackouts is not random and has a universal pattern that is independent of the disruption cause, the post-disaster network structure, and the detailed repair strategy. Our study reveals that suppressing blackout intensity is a promising way to speed up restoration.

## Abstract (web summary)

Here the authors, by using data from three electrical companies in the USA, find that the recovery duration of an outage is connected with the downtime of its nearby outages and blackout intensity and present a cluster-based recovery framework.

## Introduction

Power outages are becoming increasingly prevalent as a consequence of extreme weather patterns, aging power systems, and surging electricity need^1^. In 2020, economic loss driven by reported disasters in the US has doubled the previous average, ranking to a historical 95 billion dollars^2^. Most previous research focuses on preventing disruptions, especially cascading failures^3–8^. However, reliability itself is not enough for maintaining system resilience. Some methods proposed for suppressing outages could even increase the risk of large blackouts^9–12^. Recoverability, the ability to restore service quickly after disturbances, is as crucial as reliability in resilience.

Recovery research in power grids is usually topology-based^12–15^, and is best performed for transmission grids, where the system structure is easy to capture. These findings, unfortunately, cannot be extended to the distribution grid, which is designed and operated quite differently from meshed transmission grids. The distribution grid is a tree-like network, with the trunk at the power substation and leaves at the customers^16,17^. Small outages upstream could lead to the failure of all components downstream. As a result, the distribution grid is more vulnerable, accounting for the majority of failures in power systems^18,19^.

The recovery of power distribution systems has attracted considerable attention from the engineering community. A number of studies focused on developing optimal resource scheduling mechanisms to obtain effective restoration plans^20–22^. Methods such as routing repair crews, switching breakers, and adjusting voltage and frequency set points were adopted to reduce outage duration for customers. Outage duration reflects the direct service quality experienced by customers, the estimation of which is critical yet difficult. Multiple factors, including weather conditions^23–25^, outage locations^26^, and outage causes^24,25^ are reported to impact the recovery process. Given the complexity and opacity of variables involved in estimating restoration times, the role each factor plays in recovery remains to be explored^27^.

Compared with the considerable engineering advances, relatively little work has been done on outage restoration from a network science perspective. One of the hurdles preventing the attempts is the lack of detailed recovery data. Indeed, acquiring high-resolution and large-scale recovery data remains a challenge for further research in the field. Though large blackouts are not uncommon^6,28–30^, when and where they occur is largely unpredictable. Outage monitoring necessitates a long-term tracking that also requires fine-grained updates (at least to the magnitude of minutes). Unlike transmission outages, which are reported to government agencies such as North American Electric Reliability Corporation (NERC), information about distribution outages is shared differently by each utility, if at all. Consequently, most of the time, studies are focused on a single blackout or small region scenarios^19,31–33^.

Aside from obtaining data, modeling the outage recovery behavior poses three challenges: (i) Factors that influence the recovery speed are largely unknown, (ii) the grid condition varies on a wide range of areas and time scales, and (iii) computing data with multi-dimensional parameters can pose the problem of *curse of dimensionality*^6^. These challenges have made most previous research on smaller scales and cascading dynamics inapplicable. Similar hurdles exist in the recovery study of the broader context of complex networks, including transportation systems^34^, communication networks^35^, and financial markets^36^.

Here, we study recovery characteristics by examining real outage and recovery data from both major blackouts and daily operations across the United States. The dataset, introduced in ref. 37, offers the most complete and granular source of information on the recovery of power distribution grids. We find that the restoration duration of an outage correlates with the downtime of its nearby outages and the intensity of an blackout, but is independent of the number of customers affected. Moreover, we reveal the existence of intrinsic recovery behavior of distribution grids that is independent of microscopic weather and network details, which can be well predicted by a proposed depolymerisation-like model. These results can help make proactive responses in face of coming extreme weather and develop efficient mechanisms for speeding up recovery.

## Results

### Factors affecting recovery duration

**Fig. 1:**
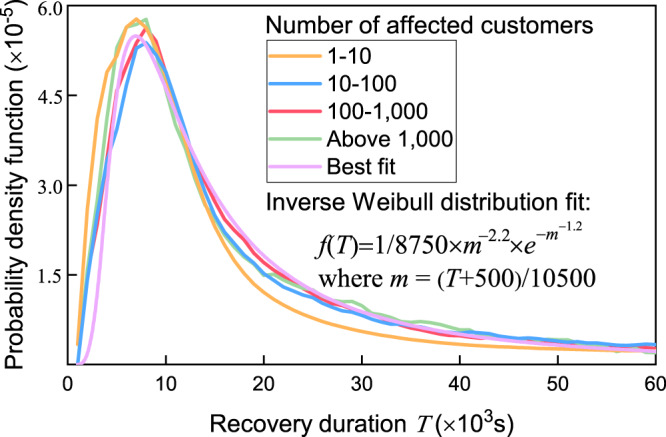
Probability density function of recovery duration. Four groups of outages with different number of affected customers are plotted. The similarity of the four curves, fitted by a universal inverse Weibull distribution function, illustrates the independence between the outage’s recovery duration and the number of affected customers.

From November 2018 to April 2020, we tracked outage reports from three electric companies in three US states (Massachusetts, New York, and Texas). The long-term tracking has recorded 682,733 outages in total, mostly from distribution grids, making the dataset ideal for the research of recovery (details are in Methods and Supplementary Note 1). Each “outage” represents the power interruption in a given area or a section of the power grid caused by component failures, such as failed substations and overload circuits. Customers without power within a service area are called affected customers of an outage, the number of which varies from fewer than 5 to over 13,000. We tested the impact of customers affected by outages and found that the recovery duration of outages is independent of the number of affected customers. We also found that the probability density function can be well fitted by an inverse Weibull distribution (Fig. 1). Accordingly, our analysis uses outage numbers instead of affected customer numbers as the metric for evaluating the extent of damage caused by a blackout. Note that the number of affected customers is a useful blackout metric related to the power loss and the system average interruption duration index (SAIDI)^38^.

**Fig. 2:**
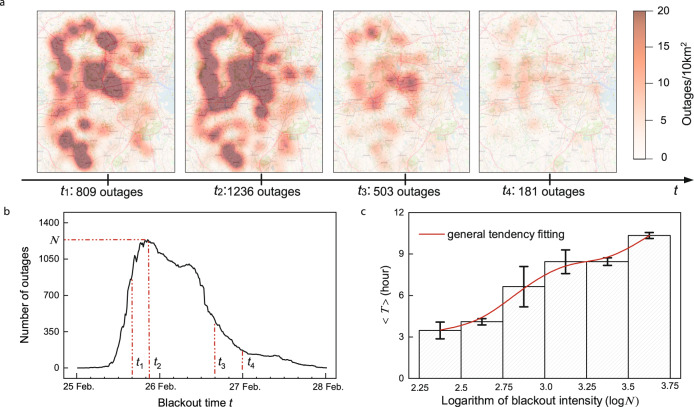
The impact of blackout intensity on recovery. **a** Illustration of the spatiotemporal evolution of a blackout on 25 February 2019 in Massachusetts. Red regions represent outages. Heatmap reproduced using OpenStreetMap and QGIS software. **b** Number of outages during the evolution of the blackout in **a**. At time *t*_2_, the outage number reaches its peak value, which is defined as the blackout intensity (denoted as *N*). **c** Statistics of the average hours 〈*T*〉 needed for a outage to recover during blackouts with different blackout intensities. The error bars show standard deviations of given blackout intensity groups. The fitting curve is obtained by general B-Spline. The interval of each group is half-open. Blackouts used for calculation are tabulated in Supplementary Table S1.

For the purposes of this analysis, we define a blackout as a collection of many outages within the same state/time frame (details in Methods), an example of which from Massachusetts beginning on 25 February 2019 is shown in Fig. 2a. All red shadows, defined as outage clusters, represent a group of outages that are physically near to each other, with color depth denoting the number of outages within. Small outage clusters merge into larger clusters as failures spread (from time *t*_1_ to *t*_2_), and can also shrink into smaller ones when recovery dominates (from *t*_2_ to *t*_4_). The total number of outages recorded varies with the size of those clusters, as shown in Fig. 2b. At time *t*_2_, the overall number of outages reaches its highest value *N*, which we define as blackout intensity. The blackout intensity reflects the peak level of damage caused by a disruption^12–14^, and is a metric that can be captured for different blackouts (Supplementary Fig. S1). By using blackout intensity, individual analysis for diverse disruptions can be simplified, thus helping recovery study.

We evaluate the impact of blackout intensity on recovery by analyzing the average time needed for outages within a blackout to get fully recovered. As depicted in Fig. 2c, the repair time needed climbs with the logarithm of blackout intensity *N*. The upward trend suggests that, as damage accumulates, more time-consuming and costly repairs are needed, with the pace of repair limited by availability of spares and repair crews. We note that there tends to be a logarithm-like dependence between blackout intensity *N* and average recovery hours *T*.

**Fig. 3:**
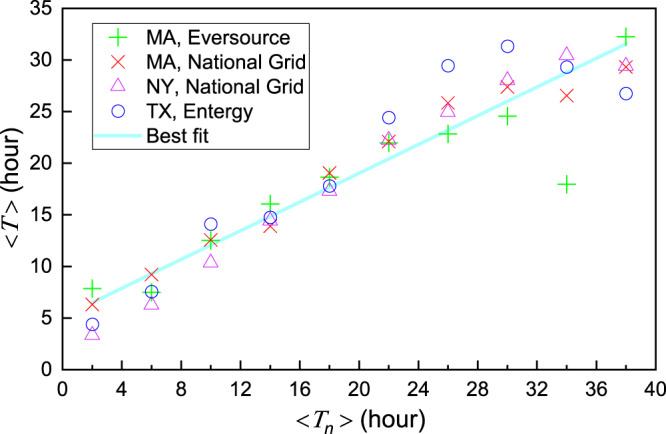
Correlation between outages and their nearby outages on recovery duration. The average recovery duration of outages 〈*T*〉 versus that of their geographically nearest *n* outages 〈*T*_*n*_〉. The data are taken from four blackouts across the United States. Specifically, two MA blackouts that began on 25 February 2019 are reported from Eversource and National Grid, respectively; the NY blackout occurred a day before MA blackouts, and the TX data was recorded by Entergy on 25 October 2019. Without loss of generality, we set *n*  =  5 as the results are insensitive to the choice of *n* (Supplementary Fig. S2).

The restoration of outages is different from the repair of failed components. Repairing one component fixes some but not all outages downstream because another downstream component failure can keep the power-loss state until all potential failures are resolved. In other words, a single outage can be caused by multiple component failures and one component failure can influence the recovery of multiple observed outages. An outage’s restoration is thus correlated with its ambient outages. Here, “ambient” does not mean that the distance between two outages has to be lower than a given threshold. As long as the two outages are connected to one damaged component, the distance between them can vary widely. Recovery of outages is also impacted by the characteristics of their surrounding infrastructures such as the type of feeder, device age, and whether there are looped connections^26^. Moreover, repair crews are likely to choose to repair nearby outages at the same time, for logistical reasons. These behaviors together indicate that the recovery duration of nearby outages is interrelated. A mapping between duration of outages and their nearby outages supports this hypothesis (Fig. 3). For each outage *i* with power loss duration *T*_*i*_, we count the average recovery time of its spatially nearest *n* outages (denoted as 〈*T*_in_〉). Dividing outages into groups based on their 〈*T*_in_〉 and calculating the mean of *T*_*i*_ (denoted as 〈*T*〉) for outages in each group (represented by 〈*T*_*n*_〉), we find a positive correlation between 〈*T*〉 and 〈*T*_*n*_〉. The positive relationship found here in different states and different companies suggests that dependence between neighboring outages is an intrinsic property of distribution grid recovery, independent of the microscopic interconnection details that change from state to state and from company to company. This finding also suggests the existence of a universal recovery model for different distribution grids.

### Cluster-based modeling of outage recovery

The detailed real-time topology of a power distribution grid would facilitate the recovery analysis, but is typically unavailable for research, and often an accurate map is unavailable, even for power grid companies, due to frequent structure changes, sparse monitoring devices, and safety concerns^39,40^. Given this, we develop a theory that does not require a specific physical topology by using the dependence we found between neighboring outages during recovery. Representing outages with nodes and linking any two nodes who have dependence (if known), we could ultimately get a cluster with all outages connected. Thereby, outage recovery can be modeled as a cluster fragmentation process. When a failed component in distribution grids gets repaired, the triggered dependence vanishes and corresponding links are removed. If sufficient links are dropped, the initial cluster would decompose into smaller ones. Possibly, we could get a small cluster including only one stand-alone node that has no link to others, which means that all the unfavorable factors preventing the outage from recovery are removed and the outage gets repaired. By iterating the process, we will eventually arrive at the stage where all links are cleared, indicating all outage repairs are complete. The system can then go back to its normal working state. Here, we consider the breakup of dependence between outages instead of directly resolving outages because as discussed above, typically there is no one-to-one mapping between outage repair and component failure repair. The fixing of one component does not mean that outages will be restored simultaneously. Instead, it indicates that a drawback deterring those outages from fixing is overcome, which can be represented by the break of dependence.

**Fig. 4:**
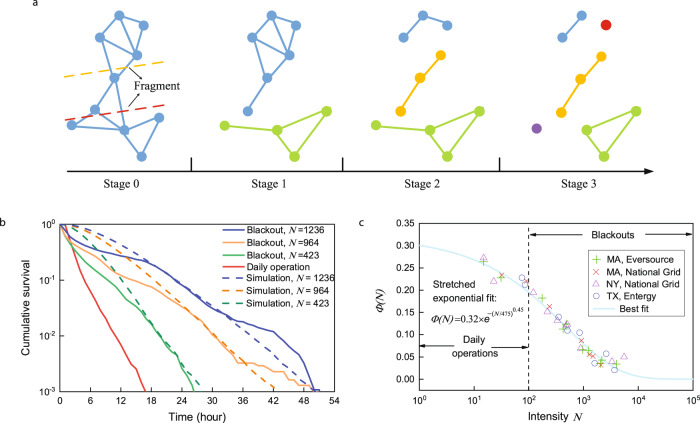
Schematic dynamics and simulation results of the cluster-based recovery framework together with the comparison of real data. **a** Illustration of a cluster fragmentation process during outages' restoration. Each node represents an outage and each link denotes the failure dependence between two nodes. At stage 1, the initial cluster breaks into two smaller clusters. One in blue with 6 nodes and the other one in green with 4 nodes. The blue cluster, with its larger size, has a higher fragmentation rate than the green one and breaks into two 3-node clusters at the next stage. At state 3, two individual nodes (red and purple) lose all of their failure dependent links, thus get repaired. **b** Survival function of outages for three observed blackouts and a month record of daily operation. Exponential distribution can be accepted by all the four curves. During the simulation, the positions of outages are the same as real data. For each blackout, 1000 realizations are simulated. **c** Illustration of a stretched exponential fit to the empirical function *ϕ*(*N*) with respect to the blackout intensity *N*. Blackouts and daily outage events used for estimation are given in Supplementary Tables S1 and S2, respectively.

The whole cluster fragmentation process above is analogous to depolymerization in materials science^41,42^. In the polymer degradation process, a widely considered model is that any chain-shaped polymer would breakup with a rate that depends on its length. A *k*-length chain can randomly cut one of its bonds and breakup into two individual chains. Although bond-cutting is random along any given chain, the overall degradation is non-random since different chains break with probabilities proportional to their lengths^41^. We extend this model to 2D plane by defining the bond of chains to be links between two sub-clusters they connect. A network visualization based on this fragmentation is illustrated in Fig. 4a. Note that links are not physical connections, but represent the dependence between outages, with a topology that is different from the tree-like structure of power distribution grids. The dependence can result from the physical structure (connection to the same failed/protected component), infrastructure characteristics (e.g., overhead or underground feeder), or the order of repair by utility crews. Undirected dependence links, modeled as Fig. 3, show that an outage will not only have impact on, but it is also influenced by its neighbor outages. When breaking the initial 10-node cluster into two parts with size 4 and size 6, we adopt a partition line that is perpendicular to the segment between the two furthest nodes in the network. In this case, there are two ways to get expected sizes for the two sub-clusters as shown by the dashed red and orange lines. If the red line is adopted, the initial cluster breaks three links and fragments into a 6-node blue cluster and a 4-node green cluster. With recursive fragmentation, five clusters occur on stage 3 with two of them (red and purple dots) containing only one node.

When this cluster-based recovery framework is applied to power distribution grids, we predict that, theoretically, the survival function of outages in each blackout follows an exponential distribution with an exponent −2*ϕ*(*N*) (Methods), which coincides well with what we found from real data (solid lines in Fig. 4b). Here, *ϕ*(*N*) is a function of *N*, representing the impact of blackout intensity on recovery. By observing 27 blackouts and 8 daily outage events across the United States, the form of *ϕ*(*N*) is fitted in Fig. 4c. It shows that *ϕ*(*N*) can be well approximated by a stretched exponential function, with an upper bound 0.32 when *N* tends to one. The upper bound shows the fastest repairing rate a power distribution grid could have. When the blackout intensity augments, recovery speed gradually decreases, which elucidates why the average duration of outages would increase when facing larger damage (Fig. 2c). In addition, *ϕ*(*N*) is sensitive to the change of blackout intensity when *N* is between 100 and 1000. Most blackouts we observed fall in this interval, meaning that suppressing blackout intensity can dramatically boost recovery. Besides, *ϕ*(*N*) equals zero when *N* is infinite, which is in line with the expectation that the time needed to repair an infinite number of outages would be infinite.

It is worth noting that the four sets of outage data in Fig. 4c are reported by different companies serving different geographical regions of the US and are caused by distinct disruptions happening at different times. Given the well-fitted stretched exponential function and that the four datasets can come from the same distribution (Supplementary Note 3), we find that there is a universal recovery pattern behind distribution networks. The pattern is determined by blackout intensity but independent of exogenous factors such as the specific causes of blackouts, post-disaster network structure, and companies’ repairing strategies.

To further test our theory, we perform a simulation of the proposed cluster-based recovery framework and compare the results with real data. As shown in Fig. 4b, the exponential decay can be well predicted by the proposed cluster-based recovery framework and coincides with real blackout performance. Despite the good agreement, we note that the exponential restoration holds only for a fraction of the entire dataset, that is, the medium part of the empirical distributions. The deviations could come from two reasons. First, in the initial stage, the existence of small outage clusters without connections to the giant component could result in the deviation from simulated results. Those small clusters, having a higher probability to generate stand-alone nodes, will skew the theoretic exponential decay to a faster speed. Second, in the final part of the restoration, the statistics of outages with long durations are sparse, which may result in deviations from our theory. These deviations can impact the exponent, but are of little significance for the present comparisons. Because our main goal is to investigate the dependence between outages during recovery, we will concentrate on cluster-based behavior. The relative flat decreasing trend at the initial stage of the simulated curves lies in that there is only one cluster at the beginning. The fragmentation of the initial cluster to generate size-one clusters takes some time since there is no preference to generate small-size clusters like what real-world polymer fragmentation does^42^.

**Fig. 5:**
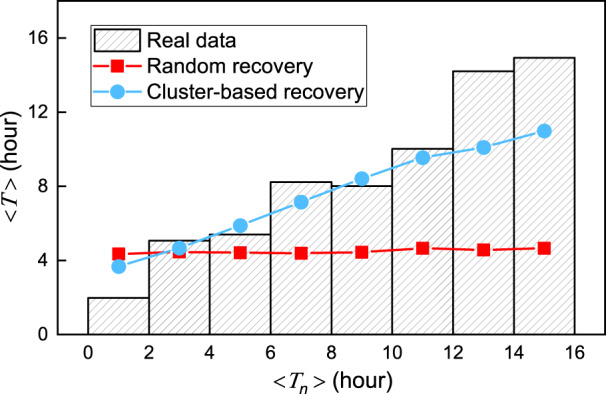
Comparison between the proposed cluster-based recovery, random recovery, and real data. The average recovery duration of outages 〈*T*〉 versus that of their geographically nearest *n* outages 〈*T*_*n*_〉. The histogram is obtained from real outage data of a blackout that began on 25 February 2019 in Massachusetts. Each bar represents the average duration of outages whose nearest *n* outages' mean recovery time fall in the given duration interval. We set *n*  =  5 as the results are insensitive to the choice of *n* (Supplementary Fig. S2). The curves shown are corresponding simulation results of random recovery (red) and proposed cluster-based recovery (blue).

We note that while randomly selecting an outage to repair with a constant repair rate of 2*ϕ*(*N*) also results in an exponential distribution, it does not capture the association with nearby outages as depicted in Fig. 3. Specifically, for random recovery, the center outage’s shutdown time remains flat as the duration of nearby outages increases (Fig. 5), while our proposed cluster-based recovery framework reproduces well the nearby outage influence found from real data.

## Discussion

Using real data from three power distribution companies in three states of the United States, we have examined the factors that impact the recovery of outages in power distribution grids. Our findings show that the restoration speed of an outage is not only determined by the overall scale of the damage in the network (blackout intensity), but also by the number of outages nearby. Although we might expect outages that affect a large number of customers have priority in recovering, the recovery duration of an outage is actually independent of its affected customer number and its probability density distribution can be well approached by an inverse Weibull distribution function. Therefore, we use the number of outages instead of affected customers in recovery analysis and find a contracting process of outages during restoration. To characterize the phenomenon, we have adopted an analytical framework based on the polymer fragmentation model. In addition, a large-scale analysis of data from multiple blackouts reveals that the recovery speed of a blackout is an inherent behavior that is only determined by its intensity, potentially independent of microscopic weather, repairing strategies, and network details. This suggests that outage recovery can be accelerated by limiting blackout intensity. Methods that could break the underlying recovery dependence between nodes in advance to prevent large blackouts could provide a promising future for recoverability enhancement. The breaking of the dependence might be achieved by updating the energy supply devices of a block. The robustness-strengthened block can thus impede neighboring blocks from generating a large outage cluster.

Blackout intensity is used in the study to quantify the impact of disruptions. The intensity itself is a compound result of multiple factors such as weather, the expanse of systems, and maintenance activities. A function reflecting the impact of those factors on blackout intensity is of vital importance, yet pretty hard to capture. Survival models like the Cox proportional hazard model^43^ might be a good alternative for mapping. If such a mapping could be established, engineers and maintainers of power grid companies can carry out risk-informed preparations and investments, to some degree, suppressing possible blackout intensity in advance.

In the proposed cluster-based recovery model, we assume there is failure dependence between outages and get an exponential survival function of outages, which agrees with the empirical data. The dependence is compounded by multiple factors, such as connection in road network, cause of damage, and available repairing resources. Although the theory fits well with real data, how could all the factors be mapped to build failure dependence links remains to be explored. A possible way is using the interdependent network. In an interdependent network, full or partial interdependence connects two adjacent layers^44^, either directed^45^ or undirected^37^, with each layer representing one related factor. In this way, the coupling strength between any two outages could be obtained and used as a metric to determine the failure dependence. Moreover, interdependence analysis can also provide insights for reducing failure dependence in power grids and preventing the formation of large outage clusters, thus speeding up the recovery process.

For realistic blackouts, our results suggest the existence of an underlying fragmentation process of outage clusters during blackouts. It is assumed in the theory that an outage cluster decomposes at a rate proportional to its current cluster size and a function of blackout intensity. The function obtained from real data fits well with a stretched exponential function, indicating a universal recovery behavior of blackouts that is independent of microscopic factors such as disaster intensity, post-disaster network topology, and blackout position. When the spreading of outages during the initial blackout process is considered, a revision of the cluster-based recovery framework is needed. The fragmentation-only model needs to be extended to a more general aggregation-fragmentation form^42^ to reflect the routine time-overlapped outage and restore processes. The aggregation simulates the propagation of failures, the dynamics of which may be modeled by a Poisson process^46^.

Instead of focusing on recovery coupling between different networks^37^, our study calls attention to the underlying dependence between different components within the power distribution grids. It indicates that a deeper understanding of recovery dependence to various contextual variables is necessary to capture the possible physical roots and thus to improve the overall efficiency of power market management and operation. It also provides circumstantial evidence that although priority-based recovery strategies can minimize the economic losses of important customers, the price is paid by the rest of the customers as the distribution of recovery duration would not change once the blackout intensity is set. The inequality from a social perspective worth further attention and study.

The power distribution grid is only one type of distributed networks that face large-scale damage. Systems such as distributed edge computing network^47^ and device layer of industrial Internet of Things^48^ face similar dilemmas as networks become increasingly complicated. Our study could be extended to other distributed networks, inspiring further collaborations between a wide range of academic and industrial fields.

## Methods

### Outage dataset

Data used in this study is recorded from publicly available websites published by three electricity companies in the United States: National Grid, Eversource, and Entergy. Data from National Grid collects outages that happened in Massachusetts and New York, while data collected from Eversource and Entergy cover the area of Massachusetts and Texas, respectively.

The outage dataset comprises a total of 682,733 outages from November 2018 to April 2020. Each outage sample contains detailed information such as the time when the loss of power is reported, time when the outage is restored, the number of customers affected, latitude, and longitude. The accuracy of each sample is one minute in time. Although samples during daily operations take up most of the data, tens of large blackouts (i.e., blackout intensity > 100 outages) are recorded, which accounted for roughly 0.05% of the total observed outages. Supplementary Note 1 shows the detailed information of the datasets used, with Supplementary Table S1 listing the identified blackouts used in this study.

### Definition of “blackouts" vs. “outages"

Blackout describes the condition when an entire region (state-level) is suffering power loss, while outage depicts the out-of-service in a given area resulting from equipment failures. Those failures can either be caused by direct nodal or line damage or indirectly by failures transmitted from upstream. Depending on the extent of the damage, an outage, defined by the live update lists of outages published by the utilities, can affect a single house or an entire neighborhood. The coordinates of an outage in this work are the coordinates published by the utilities on their maps and correspond to the center of the area without power due to the outage. Therefore, a blackout contains multiple power outages appearing and vanishing during its duration. Within a blackout duration, the number of outages will go up from a low number to a peak (blackout intensity, refer also to Fig. 2b) and then go back to its normal state. In this article, we only consider blackouts with at least 100 outages. Otherwise, the event belongs to daily operations. Some recorded blackouts and daily outage events are tabulated in Supplementary Tables S1 and S2. Unless otherwise stated, the start/end time of a blackout is 12 am on the given day.

### Cluster-based recovery process

For a given cluster with an initial size *N* (blackout intensity), the distribution of cluster size evolves as:

1 $$\frac{{{{{{{{\rm{d}}}}}}}}{S}_{k}}{{{{{{{{\rm{d}}}}}}}}t}=-{S}_{k}(t)\mathop{\sum }\limits_{i=1}^{k-1}{F}_{i,k-i}+2\mathop{\sum }\limits_{j=K+1}^{N}{S}_{j}(t){F}_{k,j-k}$$

where *S*_*k*_(*t*) is the number of outage clusters of size *k* at time step *t* and *F*_*i*,*k*−*i*_ represents the intrinsic rate that a size *k* cluster breaks up into a size *i* cluster and a size *k* − *i* cluster. The first term on the right-hand size specifies all possible breakups of a size *k* cluster, and the second term accounts for the new generations of clusters with size *k* from the collapsing of larger clusters, where the factor of 2 indicates that either of the two sub-clusters can be of size *k*.

We have already shown that blackout intensity and nearby failures influences recovery speed. Without loss of generality, let us assume *F*_*i*,*k*−*i*_ = *ϕ*(*N*)*k*, in which the fragmentation rate of a cluster depends on its own size and a function of blackout intensity it is suffering. Here, we adopt *k* instead of a function of *k* because a simple closed-form solution can already be obtained by iteration in the following. It is also in accord with the corrected Akaike information criterion (AICc)^49^, since introducing more variables into *F*_*i*,*k*−*i*_ would only add the complexity for analysis.

Given the initial cluster distribution *S*_*k*_(0) = 1 only for *k* = *N*, a closed-form solution can be obtained through iteration of (1) as follows

2 $${S}_{k}(t)=\frac{N}{k}{e}^{-\phi (N){k}^{2}t}({e}^{\phi (N)kt}-{e}^{-\phi (N)kt}),\,{{{{{{{\rm{for}}}}}}}}\,1\le k < N.$$

Detailed derivation process are shown in Supplementary Note 2. Note that when *k* = 1, *S*_1_(*t*) represents the number of repaired outages as a function of time. The unrepaired fraction of outages (*U*(*t*)) has the form of

3 $$U(t)=1-\frac{{S}_{1}(t)}{N}={e}^{-2\phi (N)t}.$$

That is, the original *N* outages disappear exponentially fast in time and the exponent is proportional to *ϕ*(*N*).

## Supplementary information

Supplementary Information

## Data Availability

The data associated with this research is available on GitHub at https://github.com/Hughie-HaoWu/Outage-recovery.

## Code availability

The code used for data analysis is available on GitHub athttps://github.com/Hughie-HaoWu/Outage-recovery^50^.
