# Supplementary material for: Fragmentation of outage clusters during the recovery of power distribution grids
Source: Nat Commun. 2022 Nov 30;13:7372. doi: 10.1038/s41467-022-35104-9 (PMC9712383; doi:10.1038/s41467-022-35104-9)
Supplement: Supplementary file 1 — Supplementary Information [file 41467_2022_35104_MOESM1_ESM.pdf]

# Supplementary Information

# Fragmentation of outage clusters during the recovery of power distribution grids

Hao Wu[1,2], Xiangyi Meng[2], Michael M. Danziger[2], Sean P. Cornelius[3], Hui Tian[1] and Albert-László Barabási[2]

[1]State Key Laboratory of Networking and Switching Technology, Beijing University of Posts and Telecommunications, Beijing 100876, China.

[2]Center for Complex Networks Research, Department of Physics, Northeastern University, Boston 02115, USA.

[3]Department of Physics, Ryerson University, 350 Victoria Street, M5B 2K3, Toronto, Canada.

**This file include:**

Note 1 to 3

Table S1 to S3

Figs. S1 and S2

References for supplementary information

## Supplementary Note 1. Outage data

The outage data comes from four open-source websites with real-time outage reports provided by Eversource, National Grid, and Entergy, three electric companies in the United States. The four websites are listed below.

- https://outagemap.eversource.com/external/default.html (Dataset 1: MA, Eversource)
- https://outagemap.ma.nationalgridus.com/ (Dataset 2: MA, National Grid)
- https://outagemap.ny.nationalgridus.com/ (Dataset 3: NY, National Grid)
- https://www.entergy.com/view-outages/ (Dataset 4: TX, Entergy)

We have recorded a total of 682,733 outages that affected 19,384,168 customers through more than a year's monitoring. The shared information they have includes start and end time, the number of customers affected, latitude and longitude, county, as well as block and its population.

Dataset 1 covers 78,512 outages in Massachusetts from 2018/11/20 to 2020/04/29. It is the only dataset among the four we obtained that does not include the cause of failure. Datasets 2 and 3 have the same monitored time period as dataset 1 does. Dataset 2 is also from MA but reported by National Grid, which has totally different power distribution systems than Eversouce does. Dataset 3, although provided by National Grid, contains data from NY. In total, 48,441 and 131,563 outages are recorded by the two datasets, respectively. Entergy data lasts from 2019/07/13 to 2020/08/12, including 424,217 outages.

All datasets contain two parts: daily data and blackout data. The daily data includes outages that often appear as individual or small-scale events that could be repaired within a short time period (usually less than 24 hours). The blackout data includes outages happening during the lifetime of blackouts, which accounts for roughly 0.05% of the total observed outages. Blackouts discussed are presented in **Table S1**. In the table, the start and end time of a blackout are both calculated at 12 o'clock in the morning

**Table S1. Recorded blackouts for analysis**

| No. | State | Company | Blackout duration | Blackout intensity |
|---|---|---|---|---|
| 1 | MA | Eversource | 2019/01/24 to 2019/01/27 | 423 |
| 2 | MA | Eversource | 2019/02/25 to 2019/02/28 | 1236 |
| 3 | MA | Eversource | 2019/07/23 to 2019/07/26 | 2023 |
| 4 | MA | Eversource | 2019/10/16 to 2019/10/20 | 3966 |
| 5 | MA | Eversource | 2019/10/31 to 2019/11/04 | 479 |
| 6 | MA | Eversource | 2020/02/07 to 2020/02/10 | 964 |
| 7 | MA | Eversource | 2020/03/06 to 2020/03/08 | 175 |
| 8 | MA | Eversource | 2020/04/13 to 2020/04/16 | 2136 |
| 9 | MA | National Grid | 2019/01/24 to 2020/01/26 | 239 |
| 10 | MA | National Grid | 2019/02/25 to 2019/02/28 | 1489 |
| 11 | MA | National Grid | 2019/10/15 to 2019/10/22 | 2070 |
| 12 | MA | National Grid | 2019/10/31 to 2019/11/05 | 916 |
| 13 | MA | National Grid | 2020/04/13 to 2020/04/17 | 1279 |
| 14 | NY | National Grid | 2018/11/26 to 2018/11/30 | 332 |
| 15 | NY | National Grid | 2018/12/31 to 2019/01/03 | 505 |
| 16 | NY | National Grid | 2019/02/24 to 2019/02/28 | 3310 |
| 17 | NY | National Grid | 2019/03/09 to 2019/03/12 | 218 |
| 18 | NY | National Grid | 2019/08/16 to 2019/08/20 | 888 |
| 19 | NY | National Grid | 2019/10/16 to 2019/10/19 | 121 |
| 20 | NY | National Grid | 2020/01/12 to 2020/01/14 | 479 |
| 21 | NY | National Grid | 2020/02/07 to 2020/02/10 | 5510 |

| No. | State | Company | Blackout duration | Blackout intensity |
|---|---|---|---|---|
| 22 | TX | Entergy | 2018/12/30 to 2019/01/01 | 350 |
| 23 | TX | Entergy | 2019/10/25 to 2019/10/31 | 1600 |
| 24 | TX | Entergy | 2020/01/10 to 2020/01/18 | 2588 |
| 25 | TX | Entergy | 2020/03/04 to 2020/03/07 | 508 |
| 26 | TX | Entergy | 2020/04/12 to 2020/04/22 | 3673 |
| 27 | TX | Entergy | 2020/06/25 to 2020/06/28 | 847 |

**Table S2. Daily operation for analysis**

| No. | State | Company | Duration | Intensity |
|---|---|---|---|---|
| 1 | MA | Eversource | 2019/02/12 12am to 2019/02/15 12am | 31 |
| 2 | MA | Eversource | 2019/12/25 8pm to 2019/12/17 12am | 15 |
| 3 | MA | National Grid | 2019/04/14 12am to 2019/04/17 12am | 91 |
| 4 | MA | National Grid | 2019/11/24 12am to 2019/11/25 4am | 32 |
| 5 | NY | National Grid | 2020/01/22 12am to 2020/01/23 8am | 23 |
| 6 | NY | National Grid | 2020/01/23 8am to 2020/01/24 4am | 15 |
| 7 | TX | Entergy | 2019/10/12 6am to 2019/10/13 4am | 85 |
| 8 | TX | Entergy | 2019/11/02 6am to 2019/11/03 12am | 75 |

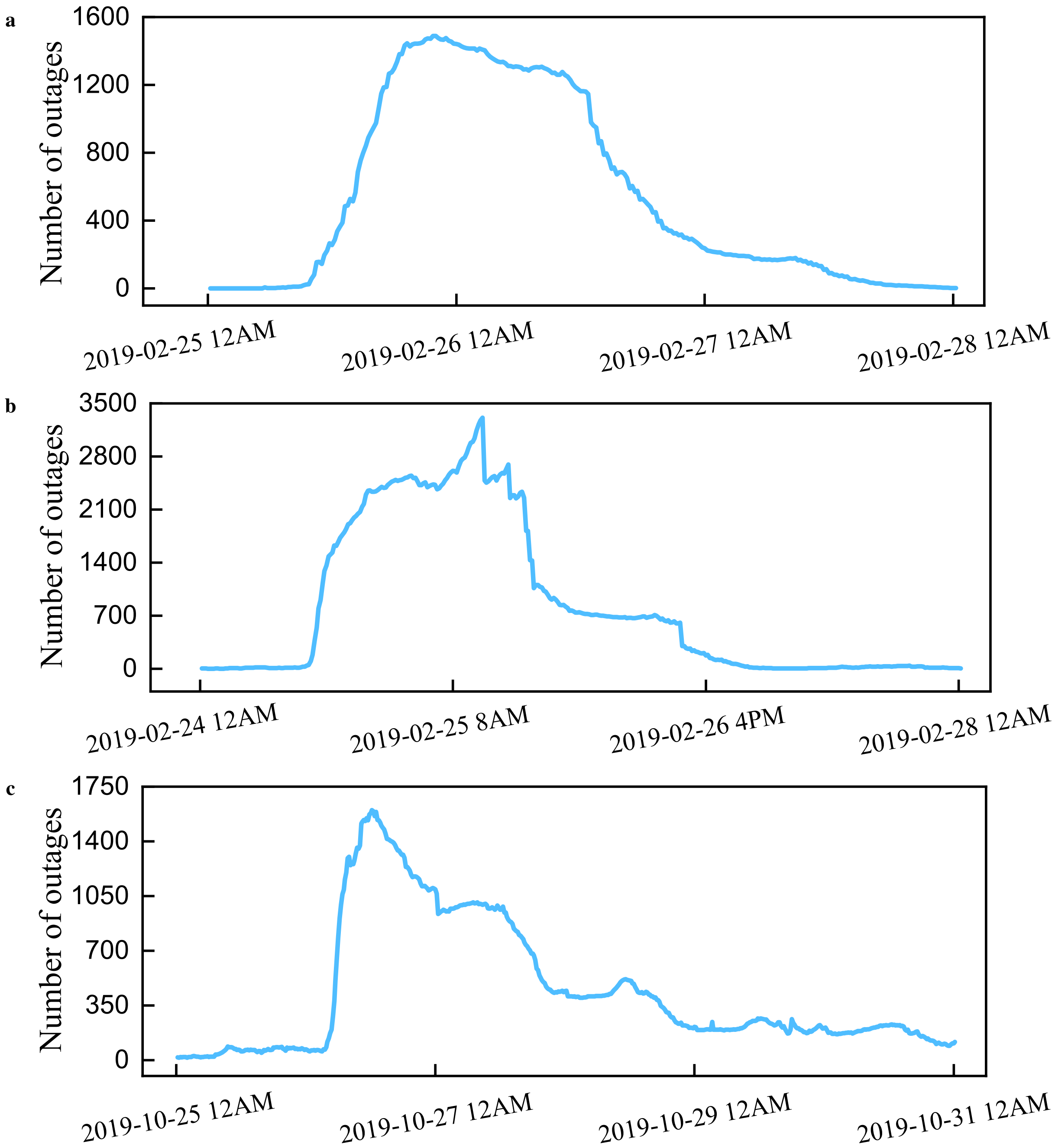


**Fig. S1.** Number of outages during the evolution of blackouts no. 10, 16, and 23 (refer to Table S1). As time goes on, outage numbers would quickly climb to a peak value and then slowly go back to a normal state.

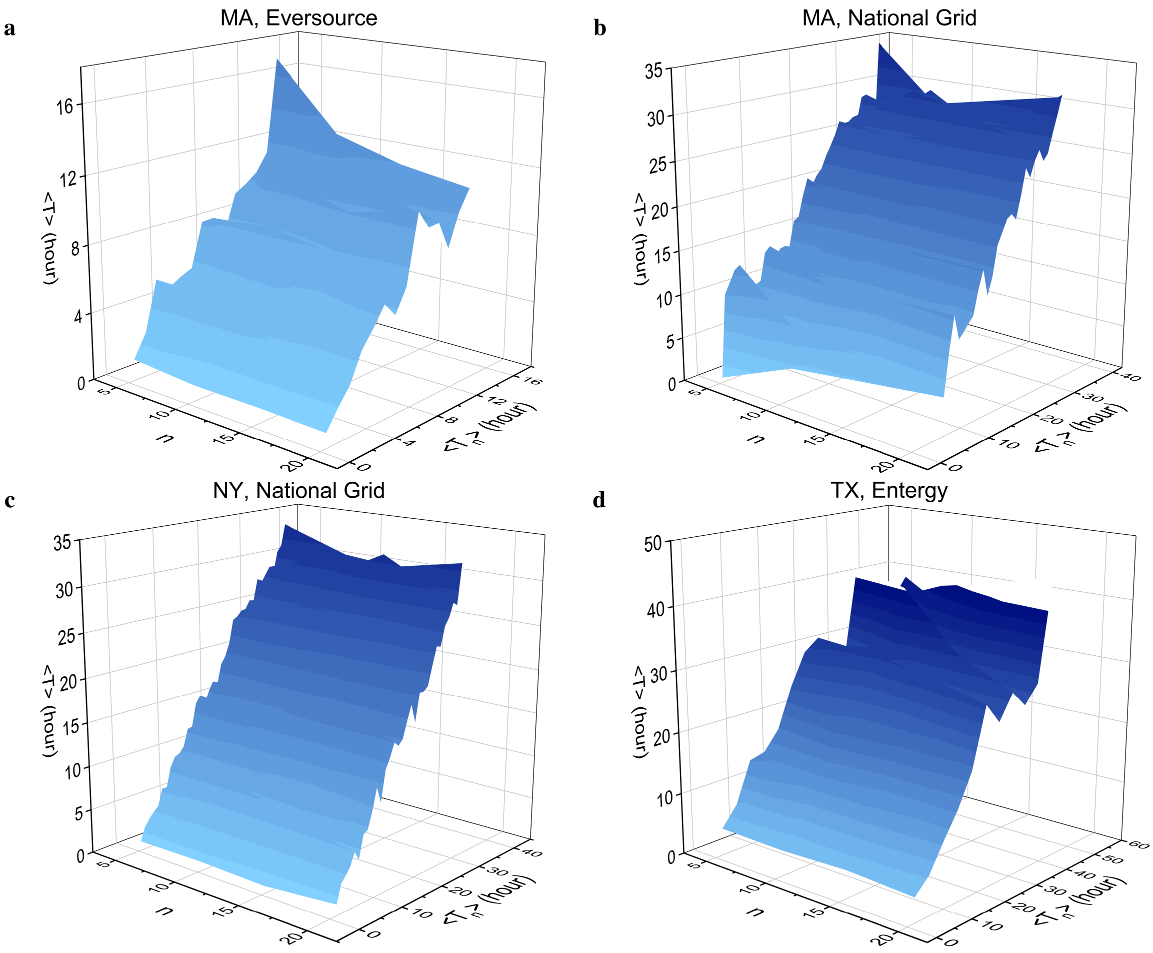


**Fig. S2.** The recovery duration of outages ($\langle T\rangle$) versus that of their geographically nearest $n$ outages ($\langle T_n\rangle$). The data are taken from four blackouts across the United States. Specifically, **a** and **b** began on February 25, 2019 in Massachusetts reported from Eversource and National Grid, respectively; **c** on a day before **b** but happened at New York, and **d** recorded by Entergy from Texas on October 25, 2019. The value of $\langle T\rangle$ has no explicit dependence on the "$n$" coordinate.

## Supplementary Note 2. Size distribution of outage clusters

For the case $k = N$, with $F_{i,k-i} = \phi(N)k$ we obtain

$$\frac{\mathrm{d}S_N}{\mathrm{d}t} = -\phi(N)N(N-1)S_N. \tag{1}$$

It implies that the probability that a cluster fragments into two sub-clusters is independent of both the sizes of the two parts. Given that only one cluster of size $N$ exists at moment $t = 0$, i.e., $S_N(0) = 1$, the evolution of cluster of size $N$ follows

$$S_N(t) = e^{-\phi(N)N(N-1)t}. \tag{2}$$

Similarly, for $k = N-1$, the kinetic equation for outage clusters with size $N-1$ becomes

$$\frac{\mathrm{d}S_{N-1}}{\mathrm{d}t} = -\phi(N)(N-1)(N-2)S_{N-1} + 2\phi(N)NS_N. \tag{3}$$

Again, at moment $t = 0$, only the initial cluster of size $N$ exists. The number of clusters with other sizes equals zero, i.e., $S_k(0) = 0$ for any $1 \le k < N$. In view of this boundary condition, by submitting (2) into (3) and leveraging the general solution of first-order non-homogeneous linear differential equation[1], we have

$$S_{N-1}(t) = \frac{N}{N-1}[e^{-\phi(N)(N-1)(N-2)t} - e^{-\phi(N)N(N-1)t}] \tag{4}$$

Continuing the iteration process for the cases of $k = N-2$, $k = N-3$, $\cdots$, we could get a general formula as follows

$$S_k(t) = \frac{N}{k}[e^{-\phi(N)(k)(k-1)t} - e^{-\phi(N)(k+1)(k)t}], \text{ for } 1 \le k < N. \tag{5}$$

This result is the number of outage clusters of size $k$, or size distribution, in the network.

**Supplementary Note 3. Two-dimensional Kolmogorov-Smirnov test (KS-test) for multiple data sources**

We have applied the two-dimensional Kolmogorov-Smirnov test to demonstrate that the recorded four sets of outage data can come from the same distribution. The two-dimensional KS-test, proposed by Fasano and Franceschini[2], is a variant on an earlier idea of Peacock[3]. It is used to test whether two samples in 2D plane (with sample size $N_1$ and $N_2$, respectively) differ. The significance level for the two-dimensional KS-test (i.e., p-value) can be summarized as[4]

$$\text{Probability (D > observed)} = Q_{KS}\left(\frac{\sqrt{N}D}{1+\sqrt{1-r^2}(0.25-0.75/\sqrt{N})}\right). \tag{6}$$

where $D$ is the maximum difference (ranging both over data samples and over quadrants) of the four corresponding integrated probabilities, $r$ is the coefficient of correlation, $N$ is the sample size with form

$$N = \frac{N_1 N_2}{(N_1+N_2)}, \tag{7}$$

and $Q_{KS}(\cdot)$ is a function defined as

$$Q_{KS}(\lambda) = 2\sum_{i=1}^{\infty}(-1)^{i-1}e^{-2i^2\lambda^2} \tag{8}$$

When the p-value is larger than 0.2, the two data samples are not significantly different and could be accepted as of the same underlying distribution[4].

As the two-dimensional KS-test can only be used for two samples. We divide blackout and daily operation data listed in **Table S1 and S2** into two groups, with Eversource data and National Grid data from NY in group A, Entergy data and National Grid data from MA in group B. Then the two-dimensional KS-test can be used to test whether two dataset distributions in each group differ. From **Table S3**, we get that the null hypothesis that the two samples in group A (B) come from the same distribution is acceptable. Therefore, we can take data in a group as one sample, and further test whether data from groups A and B share the same distribution. Again, the null hypothesis could not be rejected. To sum up, the four sets of data could be seen as from the same distribution.

### Table S3. Two-dimensional KS test results

| **Sample 1** | $N_1$ | **Sample 2** | $N_2$ | **p value** |
|---|---|---|---|---|
| MA, Eversource | 10 | NY, National Grid | 10 | 0.503 |
| MA, National Grid | 7 | TX, Entergy | 8 | 0.692 |
| Group A | 20 | Group B | 15 | 0.698 |

## References


1. Epstein, M. Partial Differential Equations: Mathematical Techniques for Engineers. (Springer, 2017).
2. Fasano, G., Franceschini, A. A multidimensional version of the Kolmogoro-Smirnov test. *Monthly Notices of the Royal Astronomical Society*, 225, 155-170 (1987).
3. Peacock, J. A. Two-dimensional goodness-of-fit testing in astronomy. *Monthly Notices of the Royal Astronomical Society*, 202, 615-627 (1983).
4. Press, W. H. *et al.* Numerical recipes third edition: the art of scientific computing (Cambridge university press, 2007).
